# A cross-sectional study to assess the clinical utility of modern visual function assessments in patients with inherited retinal disease: a mixed methods observational study protocol

**DOI:** 10.1186/s12886-023-02974-6

**Published:** 2023-05-24

**Authors:** Laura J. Taylor, Amandeep S. Josan, Irene Stratton, Jasleen K. Jolly, Robert E. MacLaren

**Affiliations:** 1 Nuffield Laboratory of Ophthalmology, Nuffield Department of Clinical Neurosciences, University of Oxford, Oxford, UK; 2 Oxford Eye Hospital, Oxford University Hospitals NHS Foundation Trust, Oxford, UK; 3 Centre for Statistics in Medicine, University of Oxford, Oxford, UK; 4 Vision and Eye Research Institute, Anglia Ruskin University, Cambridge, UK

**Keywords:** Retinitis pigmentosa, Inherited retinal disease, Rod-cone degeneration, Outcome measure, Endpoint, Microperimetry, Scotopic microperimetry, Visual acuity, Low luminance visual acuity, Moorfields acuity chart, Patient reported outcome measures.

## Abstract

**Background:**

Treatment options for patients with inherited retinal disease are limited, although research into novel therapies is underway. To ensure the success of future clinical trials, appropriate visual function outcome measures that can assess changes resulting from therapeutic interventions are urgently required. Rod-cone degenerations are the most common type of inherited retinal disease. Visual acuity is a standard measure but is typically preserved until late disease stages, frequently making it an unsuitable visual function marker. Alternative measures are required. This study investigates the clinical utility of a range of carefully selected visual function tests and patient reported outcome measures. The aim is to identify suitable outcome measures for future clinical trials that could be considered for regulatory approval.

**Methods:**

This cross-sectional study involves two participant groups, patients with inherited retinal disease (n = 40) and healthy controls (n = 40). The study has been designed to be flexible and run alongside NHS clinics. The study is split into two parts. Part one includes examining standard visual acuity, low luminance visual acuity, the Moorfields acuity chart visual acuity, mesopic microperimetry and three separate patient reported outcome measures. Part two involves 20 min of dark adaptation followed by two-colour scotopic microperimetry. Repeat testing will be undertaken where possible to enable repeatability analyses. A subset of patients with inherited retinal disease will be invited to participate in a semi-structured interview to gain awareness of participants’ thoughts and feelings around the study and different study tests.

**Discussion:**

The study highlights a need for reliable and sensitive validated visual function measures that can be used in future clinical trials. This work will build on work from other studies and be used to inform an outcome measure framework for rod-cone degenerations. The study is in keeping with the United Kingdom Department of Health and Social Care research initiatives and strategies for increasing research opportunities for NHS patients as part of their NHS care.

**Trial registration:**

ISRCTN registry, ISRCTN24016133, Visual Function in Retinal Degeneration, registered on 18th August 2022.

**Supplementary Information:**

The online version contains supplementary material available at 10.1186/s12886-023-02974-6.

## Background

Inherited retinal diseases are the leading cause of blindness in the working age population in the United Kingdom. Retinitis pigmentosa (RP) is the most common inherited retinal disease and typically presents as a rod-cone degeneration [1, 2]. These patients present with reduced night vision and peripheral visual field loss, which progresses centripetally inward and radially outward. The central vision is often spared until late disease stages. Severe visual impairment occurs when the degeneration encroaches on the central retina [2].

There are over 50 genes associated with RP and over 277 genes associated with inherited retinal disease, each with a specific disease mechanism and clinical presentation. These include the age of presentation, disease severity, rate of disease progression and sometimes syndromic manifestations [3, 4].

Currently there is only one approved treatment for a specific and rare early onset retinal dystrophy called *RPE65-*associated retinal dystrophy, this has been approved by United States Food and Drug Administration (FDA) and European Medicines Agency (EMA). There are currently no treatments for any of the other causes of inherited retinal disease, although many clinical trials are underway [2]. To ensure that meaningful outcomes of such clinical trials are captured, appropriate visual function assessments must be selected as outcome measures.

Visual acuity (VA), often referred to as Best Corrected Visual Acuity (BCVA), is the standard measure of visual function, despite it being an insensitive marker of early and subtle visual function changes in many retinal conditions [5, 6]. VA incorporates recognition of high contrast letters in high luminance conditions and only reflects the function of the central 0.5 degrees of vision, mediated by a population of foveal cones. In patients with RP, the fovea is typically preserved in early-to-moderate disease stages, so VA remains unaffected, producing a ‘normal’ result. VA only begins to deteriorate as the patient reaches advanced end-stage disease [5]. Currently, for approval of a new treatment based on VA alone, the FDA require a VA gain of 15 letters [7]. Since many patients with RP have preserved VA, many patients have little scope for further improvement, which limits the potential for regulatory approval when using this measure. These limitations are not unique to rod-cone degenerations but have also been reported for other conditions such as age-related macular degeneration and glaucoma [8, 9].

Alternative visual function measures are available and include full visual field assessments, such as kinetic perimetry. However, these tests are time consuming, tiring for patients, highly variable and in patients with RP who typically have very constricted visual fields, a lot of time is spent assessing areas with no remaining visual function [10]. Visual electrophysiology enables objective assessments of visual function. However, these techniques are complex, they require specially trained personnel, take a long time to perform and testing conditions must be carefully controlled to reduce noise in the data [11]. Full-field electroretinography testing is often insensitive to subtle changes in retinal sensitivity or not measurable in many patients with rod-cone degenerations [12]. Furthermore, full-field electroretinography provides a global retinal function assessment as opposed to a measure of localised functional changes, that is required in assessment of targeted therapeutic treatments.

Many of the standard clinical visual function measures currently employed are insensitive to changes due to novel therapies for these rod-cone degenerations [13]. Without suitable outcome measures, treatment effectiveness cannot be determined, delaying any patient benefit. This problem is not confined to inherited retinal disease but also apparent in other conditions such as age-related macular degeneration. The MACUSTAR consortium was set up to identify useful outcome measures for both clinical trials and clinical practice in age-related macular degeneration [6]. To address this need in severely low vision patients the HOVER consortium was formed [14]. Neither of these consortia addresses the problems in patients with early-to-moderate inherited retinal disease causing rod-cone degeneration.

Patients with rod-cone degenerations report difficulty in low light conditions [2], so visual function tests designed to measure function under low light levels may be more sensitive and could allow for more accurate metrics of disease progression and be more relevant to patient experiences. This study aims to investigate the clinical utility of a range of selected visual function tests and patient-reported outcome measures, to identify those suitable for use as outcome measures in future clinical trials to support regulatory approval.

## Methods

The VFIRD study is a cross-sectional prospective study involving two groups of participants: participants with an inherited retinal disease causing a rod-cone degeneration (n = 40), and age and gender matched healthy controls (n = 40). Healthy controls will be recruited from members of the general public through advertisements or from individuals accompanying patients (must be non-blood relatives) to eye hospital appointments. Patient participants will be recruited from the NHS specialist ocular genetics clinic at Oxford Eye Hospital. This publication refers to protocol version 2.1 dated 22/11/2022 and was developed using the STROBE and SPIRIT reporting guidelines where applicable [15, 16]. The study is sponsored by the University of Oxford. A patient and public involvement (PPI) group was set up with representatives providing feedback which supported the study design. Ethics approval has been obtained from the NHS Health Research Authority Black Country Research Ethics Committee (reference 20/WM/0283).

### Study outcomes

Primary outcome: To assess the utility, validity and reliability of current visual function tests as clinical and research measures, in patients with inherited retinal degeneration.

Secondary outcome: To assess the utility, validity and reliability of new visual function tests as clinical and research markers in patients with inherited retinal degeneration.

To gain understanding of patient participant experiences and feelings about these alternative tests and identify whether they are appropriate, acceptable, and accessible, or whether any changes need to be made.

### Exploratory outcomes

To gain a greater insight into genotype-phenotype correlations for specific causes of rod-cone degeneration.

### Screening

Patient participants will be screened from the NHS genetics clinic (Table 1) and invited to take part in the study a least two weeks in advance of their appointment, where upon interest in the study they will be provided with the participant information sheet. The PPI group advised that two weeks should be the minimum time to ensure participants have time to consider their enrolment and make travel plans. Control participants, upon interest in the study, will be provided with the study information sheet to decide if they fulfil the criteria (Table 1) and consider if they wish to take part.

**Table 1:** Summary of the VFIRD study eligibility criteria

Inclusion criteria
**The patient participant may enter the study if ALL the following apply:**
• Participant is willing and able to give informed consent for participation in the study
• An inherited retinal degeneration diagnosis
• A minimum of 6/60 (logMAR 1.0) standard VA in each eye
• Able to participate in visual function testing
**The control participant may enter the study if all the following apply**:
• Participant is willing & able to give informed consent for participation in the study
• Male or female, aged 16 or above, there is no upper age limit
• A minimum standard VA of 6/7.5 (logMAR 0.10) in each eye – this will only become apparent once the participant starts the study, if it is clear they do not meet this criterion, they will be excluded from the study and no further testing undertaken
• Able to participate in visual function testing
**Exclusion criteria**
**The patient may not enter the study if ANY of the following apply**:
• Pre-existing amblyopia or squint would exclude that eye, but the unaffected eye would still be eligible
• History of eye problems, eye treatment or eye surgery other than refractive error. If an eye problem exists in one eye, the fellow eye is still eligible
• Been involved in an interventional research trial where they have received a treatment for their eye condition
**Control participants may not enter the study if ANY of the following apply**:
• Pre-existing amblyopia or squint, fellow eye still eligible
• History of eye problems, eye treatment or eye surgery other than refractive error. If an eye problem exists in one eye, the fellow eye is still eligible

### Study procedures

**Fig. 1:**
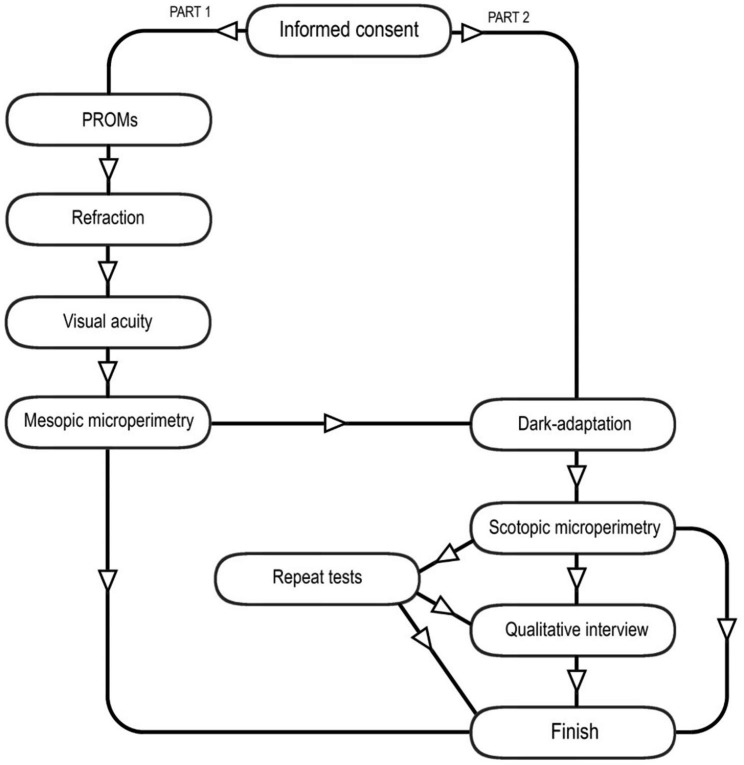
Illustrates the visual function tests included in part one and part two of the study alongside the testing order

Informed consent will be obtained, in person, by the investigator (appointed research optometrist) prior to completing any study procedures. This is a single visit study that has been carefully designed to fit in and around the NHS outpatient appointments at Oxford Eye Hospital, to minimise the need for participants to return to the eye hospital to take part in the study on a separate day. This is important as the specialist retinal genetic clinics are part of a tertiary referral centre, meaning that patients travel from all over the country to attend. The study has been planned to be as flexible as possible to work around time and room availability constraints, therefore not all the study tests are required to be completed. The study is divided into two parts, part one and part two (Fig. 1). Participants must complete all tests in a single part, although where possible participants will be invited to complete the entire study. A subset of patient participants will be invited to take part in semi-structured qualitative virtual interviews upon completion of the study. Estimated study duration is one hour for each part, two hours in total for the entire study.

### Part one

#### Patient reported outcome measure questionnaires

Three visual function and quality of life questionnaires (Visual Function Question-25, the Low Luminance Questionnaire and the EQ-5D-5L) are included in the study. To the best of our knowledge, there is little literature around the use of the Low Luminance Questionnaire and EQ-5D-5L for patients specifically with inherited retinal disease. Patients will be required to complete these, ideally before further visual function testing.

#### Refraction and acuity tests

**Fig. 2:**
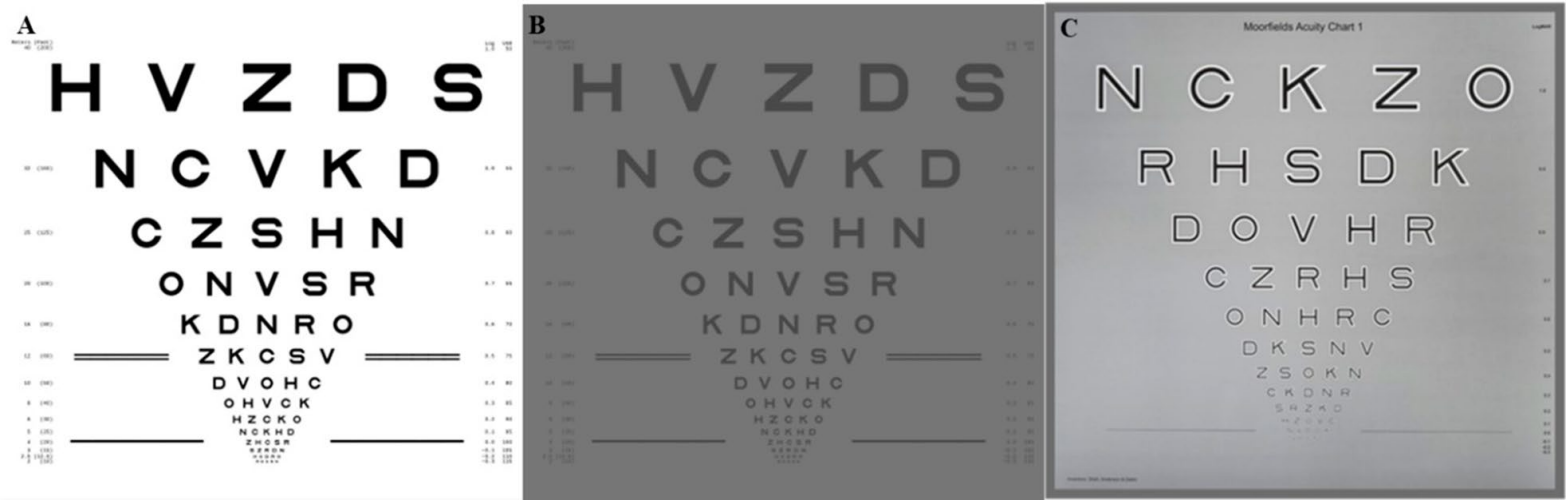
(**A**) is an example of the standard ETDRS chart. (**B**) illustrates low luminance visual acuity, simulating the view of the ETDRS chart through the 2.0 log unit neutral density filter. (**C**) is the Moorfields Acuity Chart

Refractions will be performed at 4m, in accordance with the Early Treatment Diabetic Retinopathy Study (ETDRS) Chart (Fig. 2A) and protocol [17]. This protocol standardises the assessment of VA in clinical trials and remains a prominent functional assessment in most ophthalmic clinical trials.

Low luminance VA (Fig. 2B) will be measured first following refraction, using a 2.0 log unit neutral density filter placed in front of the patient’s right eye, with the room lights switched off and using the retro illuminated standard ETDRS visual acuity chart (luminance 1.6 cd/m^2^). The none testing eye will be occluded throughout. Table 2 details the VA test, eye and corresponding letter chart (R, 1 or 2) testing order. The letter charts are rotated at each assessment to limit any letter sequence memorisation.

**Table 2:** Visual acuity, eye and corresponding letter chart testing sequence

Visual acuity testing sequence and letter chart rotation
1. Refraction of both eyes, using chart R
2. Right eye low luminance VA, left eye occluded, chart 1
3. Right eye standard VA, left eye occluded, chart 1
4. Left eye low luminance VA, right eye occluded, chart 2
5. Left eye standard VA, right eye occluded, chart 2
6. Repeat right eye low luminance VA, left eye occluded, chart R
7. Repeat right eye standard VA, left eye occluded, chart R
8. Binocular low luminance VA, chart 1
9. Binocular standard VA, chart 1
10. Left eye MAC VA, right eye occluded chart 2
11. Right eye MAC VA, left eye occluded, chart 1
12. Repeat left eye MAC VA, right eye occluded, chart 2
13. Binocular MAC VA, chart 1

The Moorfields Acuity Chart (MAC) is a modified version of the ETDRS letter chart (Fig. 2C), it has been developed to be a more sensitive measure of early changes in central cone photoreceptor disease in age-related macular degeneration. The chart uses vanishing optotypes, whereby, once the letter is no longer recognisable it is also undetectable [8]. MAC chart assessment follows LLVA and standard VA assessment, with the room lights switched on. Again, table 2 details the eye testing order and corresponding chart to use.

#### Mesopic microperimetry

Mesopic microperimetry using the MAIA (Macular Integrity Assessment; Centervue SpA, Padova, Italy) assesses central visual function, whilst using fundus-tracking technology to improve test accuracy and reliability [18]. This is a highly useful test as it produces a central threshold sensitivity map with clear and easy to interpret results indicating visual function capabilities. We have shown that microperimetry is a useful, repeatable clinical marker in other inherited retinal diseases [19, 20]. Microperimetry was a main outcome measure in the phase I/II gene therapy clinical trials for *RPGR*-associated RP [21]. Application and utility assessment are still required in other RP subtypes due to the disease’s heterogeneity. Mesopic microperimetry will be used as a marker to identify disease severity and to compare with the other visual function results.

**Fig. 3:**
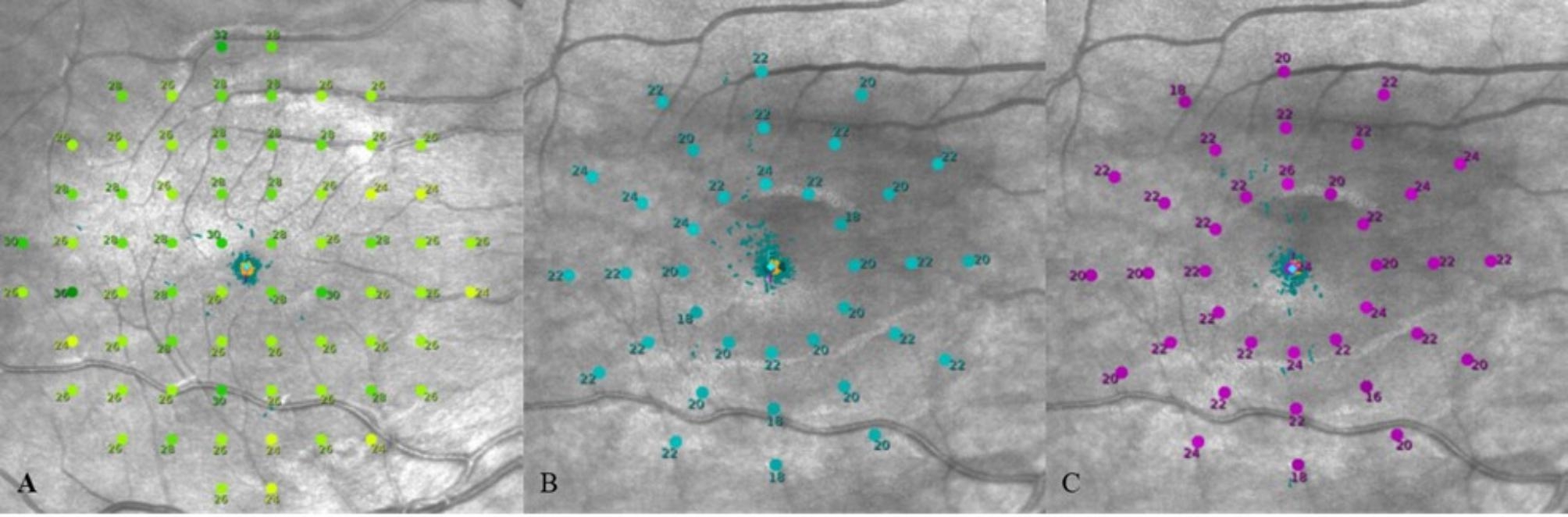
**A**, represents results from the 10-2 rectilinear testing grid used in mesopic microperimetry. **B** and **C** represent results from the radial 37 stimuli testing grid used in scotopic cyan and scotopic red microperimetry testing, respectively

Microperimetry will be performed on all participants completing part one, in a darkened room (light level < 1.0lx), without any formal dark adaptation or any pharmacological pupil dilation [22, 23]. The standard 10−2 test grid (Fig. 3A) will be used, with 4−2 bracketing threshold strategy. Examination involves the presentation of Goldmann size III stimuli of various intensities (0-318cd/m^2^), presented for 200ms, on to a mesopic background (1.27cd/m^2^). The overall dynamic testing range is 0-36dB. A 1-degree diameter red circle will be used as a central fixation target. Prior to testing, subjects will be told about the test and what they are required to do. The right eye is to be tested first followed by the left eye as per clinical convention. The non-tested eye must be occluded throughout.

### Part two

#### Scotopic Microperimetry

Scotopic microperimetry is a modified version of microperimetry, that is performed at much lower light levels, using two-colour perimetry to target a spatial assessment of rod and cone photoreceptor scotopic function [24]. For clinical trials, this test could be incredibly useful in identifying rod or cone specific photoreceptors responding to treatments. However, this test can be more difficult for patients to complete, it is prone to ceiling and floor effects [18]. Clinical utility assessment of scotopic microperimetry in patients with RP at different disease stages is vital to enable us to understand whether it is likely to be a practical and appropriate measure.

Scotopic microperimetry testing, will be performed on the S-MAIA microperimeter. Each participant must complete 20 minutes of dark adaptation (light level < 1.0lx) prior to testing [25]. The 37-point scotopic wide radial grid will be used (Fig. 3B), with a 4−2 bracketing threshold staircase strategy and Goldmann size III (0.43degrees) stimulus. Cyan stimuli testing (505nm) is performed first followed by red stimuli (627nm) testing. A 1-degree circular red fixation target (645nm) is used, if this is not seen at the minimum brightness, the luminance will be increased until the fixation target is detected. No formal break will be given between each test. A subset of patient and healthy control participants (at least 50%) will undergo repeat testing on one eye only for repeatability analyses.

#### Qualitative data collection

Patient participants who complete both study parts one and two will be invited to take part in a recorded semi-structured interview, ideally within a few days of completing the study. The aim is to assess participants thoughts and feelings around the practicalities of the different visual function tests being undertaken, as well as suggestions for improving the experience in the future. Interviews will take place over Teams at a mutually agreed time, it is estimated each interview will take around 30–45 minutes. The audio recordings from the interviews will be transcribed and used for thematic analysis.

#### Additional data

Demographic data including age and ophthalmic history from the patient participants’ hospital notes will be collected as part of the study. Structural outcome measures obtained from retinal imaging includes optical coherence tomography (OCT) and fundus autofluorescence attained using the Heidelberg Eye Explorer software (Heidelberg Engineering, Heidelberg, Germany) will be used to investigate structure-function correlations. Structural outcome measures provide objective assessments by combining structural dimensions to functional endpoints, which can improve predictive accuracy for short-term data, increase the statistical power of tests, potentially resulting in smaller sample sizes being sufficient [7, 26]. However, structural endpoints require evidence of strong correlations with functional biomarkers to be accepted as clinical trial endpoints [7, 13].

#### Safety

The VFIRD study is observational, there are no therapeutic interventions administered. All visual function tests used in the study are already CE approved and commercially available tests. All assessments are non-invasive and not associated with any serious adverse events. Some participants may find some of the testing tiring, but this has been carefully considered and protocols tailored to minimize fatigue. For patient participants, if any concerns arise during the study, these will be recorded in the patient’s hospital notes and bought to the attention of the patient’s consultant who is responsible for their care. For healthy participants, if any concerns arise regarding reduced visual function, the participants will be advised to see their optometrist or general practitioner for further investigation. Participants may withdraw at any point during the study.

### Statistical analysis

#### Study sample size

There is insufficient data on the variance of most of these visual function tests, since many have not been used in patients with inherited retinal disease and more specifically, rod-cone degeneration, to enable accurate method comparison and power calculations. The data obtained from this study will be used to estimate power and calculate sample sizes in future clinical trials. Therefore, we apply a pragmatic sample size estimation. On average 30 patients a week attend the eye hospital with inherited retinal disease.

Around 40% of patients with inherited retinal disease have RP [27]. From this we calculate 12 patients with RP attend the eye hospital per week on average. Not all will be eligible. Those with advanced disease resulting in unmeasurable vision will be excluded. We estimate 50% will be eligible. Patients are referred to our tertiary referral centre to learn more about their condition and enquire about research. From previous studies, patient recruitment rate has been good, with at least four in five patients willing to take part in research studies. We estimate there will be four eligible patient participants willing to take part each week. The project must work around other research and clinic availability.

We have allocated 15 months (65 weeks) for data collection. To avoid patient and participant inconvenience and clinic disruption, it will not be possible to test more than two participants in one day. Some weeks we will be able to complete testing for two participants (particularly if they are together), while other weeks it may be only possible to complete one. Therefore, we have set a conservative target of 1–2 participants per week. From this we generated an estimated sample size of 40 patient participants and 40 healthy volunteer participants. We believe 80 participants is achievable and will provide sufficient end-to-end data distribution for this early-stage study. Our work investigating tests in other eye disease have used similar sample sizes [19, 28].

#### Qualitative sample size

We estimate a sample size of 20–25 participants, although this is flexible. The sample size will be adjusted as deemed sufficient upon ongoing data analyses to ensure data adequacy [29]. These participants will be purposely sampled following completion of the study visual function tests. The participants will all have confirmed inherited eye disease causing similar types of sight loss (loss of night vision and peripheral vision), with variable severity.

#### Assessment of clinical utility & statistical methods

Smart’s clinical utility multi-dimensional model will be used to comprehensively explore the clinical effectiveness but also evaluate the tests ease and efficiency of use, their relevance and more importantly patient’s and participant’s perspectives [30]. The model is broken down into four aspects but there is some overlap between these:

- Appropriateness – are the tests effective and relevant?
- Accessibility – are they readily available and affordable?
- Practically – are they functional and straightforward?
- Acceptability – are they ethical and of acceptable patient burden?

To assess appropriateness and practicality of these new assessments we will apply descriptive statistics to indicate the number of participants able to complete each test. Followed by distribution plots and frequencies to indicate the spread of data. An appropriate visual function test is one that shows a large range of results across the disease spectrum with limited ceiling and floor effects. It should distinguish between healthy controls and patient participant’s vision. To investigate this, comparative statistics will be applied. The degree of difference will be analysed using Cohen’s D to calculate the effect size. ANOVA or non-parametric equivalents will be used to test for statistically significant differences, between patients and controls. The threshold of statistical significance will be set at p < 0.05. Test time will be measured and used as a quantitative marker for accessibility.

To investigate test-retest variability, a subset of participants will complete the study tests to enable Bland-Altman repeatability analyses with resulting limits of agreement and coefficient of repeatability indices. This will identify a clinically significant criterion [31]. Patient participants may be categorised by their genotype and their clinical characteristics, to develop greater understanding of genotype-phenotype correlations.

Regression analyses will be applied to compare the new visual function test results to currently used measures including VA, patient reported outcome measure questionnaires and ocular imaging structural analyses. A strong correlation would suggest tests are assessing similar aspects of the patient’s vision. This is important since it can help us understand which visual function assessments may be more relevant to specific patient reported experiences. Missing or incomplete data will be excluded from analyses.

#### Qualitative data analysis

All recorded qualitative semi-structured interviews will be transcribed manually by an approved transcriber. Thematic analysis using Nvivo software will be applied to identify common themes in data regarding participants experiences and feelings of the overall study and specific study tests. These will be summarised using network diagrams to illustrate interpretation. The aim is to explore the accessibility, practicality and acceptability of the different tests [32].

#### Data management and quality assurance

Data collected will include demographic information, details of participants’ eye conditions and ocular history, general health, results of questionnaires, vision tests results, as well as audio recordings and transcripts of interviews. Demographic data will be recorded on the clinical reporting forms. Data arising from visual function testing, such as VA, will be recorded on prepared worksheets, that make up the clinical reporting forms. Similarly, completed visual function questionnaires will also form part of the clinical recording forms. Digital data formats include spreadsheets with demographic, historic and VA results compiled from the clinical recording forms, plain text files (.txt), portable document format files (.PDF) and image files (.JPEG or .PNG) generated from microperimetry testing and retinal imaging. MP3 Audio files will be generated from the qualitative interviews. File format is determined by the output of the generating device/test. All files will use standard formats to maximise accessibility. Currently, file volume details are unknown, although they are not expected to be large.

The participants will be identified by a study specific participant number, this will be used throughout the study on all study paperwork, imaging and audio files that relate to that individual. Only the signed consent form will contain participants name and contact email. All documents and data will be sorted securely and will only be accessible by authorised personnel. The study will comply with the UK General Data Protection Regulation (UK GDPR) and the Data Protection Act 2018.

Following completion of the study and publication of the results the visual function data will be made available via the publicly accessible University of Oxford ORA-Data archive. This will be traceable with registered DOI numbers. Licensing agreements will be set up to ensure anyone who uses the data acknowledges the use by referencing the DOI.The data will be retained in this repository for as long as it is deemed valid. Apart from protecting personal data, there are no restrictions on sharing the data. The data will be organised into established file formats, e.g. .CSV files or .text files. These will be organised into folders categorised by test type; text files will include metadata details.

Study data (including personal information such as consent forms) will be stored for 3 years as per university policy, commencing from the end date of the study. This includes audio recordings (in case of needing to check audio details such as tone of voice after transcription) and transcriptions of interviews.

#### Quality Assurance

All investigators will have completed Good Clinical Practice (GCP) training. Training and delegation logs will be maintained for all those working on the study. Periodically the data will be monitored by the principal investigator to ensure accuracy and quality, the results of these audits will be shared with all investigators involved in the study and the PPI group, amendments to the study will be made where required. All study changes including protocol amendments will be shared with all study investigators and the PPI group.

#### Dissemination

Upon completion of data collection, results will be published in open access national and international journals. Results will also be presented at national and international ophthalmic conferences to ensure widespread coverage. Summaries and links to these publications will be shared on social media. All investigators will be responsible for the authorship of these publications, in accordance with the International Committee of Medical Journal Editors (ICMJE) guidelines. Press release summaries will be shared with patients, patient organisations and the general public, these will be written with input from the patient and public involvement group.

## Discussion

Identification of appropriate tools that can adequately assess changes in visual function resulting from novel therapeutic treatments for inherited retinal degenerations are urgently required. It is of paramount importance that clinical trial protocols move away from a ‘one size fits all testing approach’ using a battery of unsuitable visual function assessments and instead consider outcome measures relevant to specific disease mechanisms, clinical characteristics and the level of visual impairment. Ideally, an outcome measures framework specific for every type of inherited retinal disease is required. However, due to the large number of gene specific retinal diseases, this is impractical. Instead, by grouping conditions based on clinical presentations and type of primary degeneration, such as rod-cone degeneration, cone-rod degeneration, macular degeneration, an outcome measure framework for each group may be a more pragmatic approach. Hence the overarching aim of this project is to create an outcome measure framework for patients with early-to-moderate rod-cone degeneration.

Recent research has focused on improving visual function measures in patients with choroideremia and *RPGR-*associated RP, such as optimising microperimetry testing, visual field testing, VA testing strategies and understanding the clinical significance of low luminance VA [5, 10, 22, 23, 28, 33–35]. Further research has focused on other measures of visual function in choroideremia, including colour vision and near vision reading acuity [36, 37]. This project builds on this previous work, investigating outcome measures in a broader cohort of patients with inherited retinal disease.

Once suitable outcome measures are identified, careful consideration around the units of measurement and statistical analyses is required. A recent study recommended that volume sensitivity indices should be employed in microperimetry, as opposed to mean sensitivity [33]. In VA analysis, it has been suggested that using a comparison of mean change from baseline for the entire cohort, as an endpoint is superior to using a binary endpoint cut-off; since it maximises the information gained from the data, accounting for both worsening and VA improvement. VA reporting as a categorical variable (e.g. number of individuals shows a >15 letter change) may be more suitable as a secondary outcome, used to support letter score analyses [38]. However, natural regression towards the mean, has been highlighted as a limitation, when considering mean VA changes [39, 40].

In the United Kingdom, the government and other healthcare stakeholders have published policy documents, describing strategies to increase the breadth of research and build capacity to enable every NHS patient to have the opportunity to be involved in research [41]. This study is in line with these ambitions, it has been carefully designed to fit flexibly alongside NHS ophthalmology retinal genetic outpatient clinics. The research experience gained from this study will inform and direct future research activities.

The VFIRD study aims to address an urgent and unmet need for better outcome measure selection to optimise clinical trial design and ensure appropriate endpoints that are deemed acceptable by the regulators. This is a timely and important study that will help inform an outcome measure framework for future clinical trials for conditions specifically presenting as a rod-cone degeneration. In addition, the study will also highlight practicalities of undertaking a research study alongside an NHS clinic and provide recommendations to optimise this in future.

## Electronic supplementary material

Below is the link to the electronic supplementary material.

Supplementary Material 1

## Data Availability

Not applicable. See data management plan for plans around handling study data.

## List of Abbreviations

BCVA: Best corrected visual acuity
EMA: European Medicines Agency
ETDRS: Early treatment diabetic retinopathy study
FDA: Food and Drug Administration
GCP: Good Clinical Practice
IMCJE: International Committee of Medical Journal Editors
MAC: Moorfields Acuity Chart
MAIA: Macular integrity Assessment
NHS: National Health Service
OCT: Optical coherence tomography
PPI: Patient and public involvement
REGA: Research Governance, Ethics & Assurance Team
RP: Retinitis pigmentosa
VA: Visual acuity
VFIRD: Visual Function in Retinal Degeneration
