# Supplementary material for: A cross-sectional study to assess the clinical utility of modern visual function assessments in patients with inherited retinal disease: a mixed methods observational study protocol
Source: BMC Ophthalmol. 2023 May 24;23:234. doi: 10.1186/s12886-023-02974-6 (PMC10210467; doi:10.1186/s12886-023-02974-6)
Supplement: Supplementary file 1 — Supplementary Material 1 [file 12886_2023_2974_MOESM1_ESM.docx]

Supplementary material 1: Example of the control consent form.

**CONTROL CONSENT FORM**

**Study Title:** Visual Function in Retinal Degeneration

*Name of Researcher: If you agree, please initial box*

| 1. 1. I confirm that I understand what the research study involves and I have had the opportunity to consider the study information sheet (dated…………, version……….), ask questions and have had these answered satisfactorily. |  | |
| --- | --- | --- |
| 1. I understand that my participation is voluntary and that I am free to withdraw at any time without giving any reason, without my medical care or legal rights being affected. |  | |
| 1. I understand that the data collected during the study may be looked at and used by individuals from University of Oxford, from regulatory authorities and from the NHS Trust(s), where it is relevant to my taking part in this research. |  | |
| 1. I agree to take part in this study. |  | |
| Additional: |  | |
| 1. I would like to be provided with a copy of the results from this study | Yes | No |
|  |  |  |

| *_______________________* | *_________________* | *___________________________* |
| --- | --- | --- |
| *Name of Participant* | *Date* | *Signature* |
| *_______________________* | *_________________* | *___________________________* |
| *Name of Person taking Consent* | *Date* | *Signature* |

**1 copy for participant; 1 copy for researcher site file.*

Supplementary material 2: Examples of patient consent form

**PATIENT CONSENT FORM**

**Study Title:** Visual Function in Retinal Degeneration.

*Name of Researcher: If you agree, please initial box*

| 1. I confirm that I understand what the research study involves, and I have had the opportunity to review the study information sheet (dated…………, version……….), ask questions and have had these answered satisfactorily. |  | | | |
| --- | --- | --- | --- | --- |
| 1. I understand that my participation is voluntary and that I am free to withdraw at any time without giving any reason, without my medical care or legal rights being affected. |  | | | |
| 1. I understand that relevant sections of my medical notes and data collected during the study may be looked at by individuals from University of Oxford, from regulatory authorities [and from the NHS Trust(s)], where it is relevant to my taking part in this research. I understand that genetic mutation data and retinal images may be used in publications in a form that does not identify me. I give permission for these individuals to have access to my records. |  | | | |
| 1. I agree to take part in this study. |  | | | |
| 1. I agree to be contacted following the study for an audio recorded interview about my thoughts and feeling around the different study tests. | Yes | | No | |
|  |  | |  | |
| Additional: I agree to audio recording and the use of anonymised quotes in research reports and publications. I understand that it will not be possible for me to be identified by these quotes. | Yes | No | | |
|  |  |  | | |
| 1. I would like to be provided with a copy of the results from this study | Yes | | | No |
|  |  | | |  |

| *_______________________* | *_________________* | *___________________________* |
| --- | --- | --- |
| *Name of Participant* | *Date* | *Signature* |
| *_______________________* | *_________________* | *___________________________* |
| *Name of Person taking Consent* | *Date* | *Signature* |

**1 copy for participant; 1 copy for researcher site file; 1 (original) to be kept in medical notes* (if participant is a patient).
